# One-Pot Route towards Active TiO_2_ Doped Hierarchically Porous Cellulose: Highly Efficient Photocatalysts for Methylene Blue Degradation

**DOI:** 10.3390/ma10040373

**Published:** 2017-03-31

**Authors:** Xiaoxia Sun, Kunpeng Wang, Yu Shu, Fangdong Zou, Boxing Zhang, Guangwu Sun, Hiroshi Uyama, Xinhou Wang

**Affiliations:** 1 College of Textiles, Donghua University, Shanghai 201620, China; xxsun@dhu.edu.cn (X.S.); wang.k.peng@foxmail.com (K.W.); gool228@163.com (F.Z.); 2 Key Laboratory of Textile Science & Technology of Eco-Textile, Ministry of Education, Donghua University, Shanghai 201620, China; 3 Key Laboratory of Textile Science & Technology, Ministry of Education, Donghua University, Shanghai 201620, China; 4 Department of Applied Chemistry, Graduate School of Engineering, Osaka University, Osaka 565-0871, Japan; y_shu@chem.eng.osaka-u.ac.jp (Y.S.); uyama@chem.eng.osaka-u.ac.jp (H.U.); 5 South China Advanced Institute for Soft Matter Science and Technology, South China University of Technology, Guangzhou 510641, China; bxzhang@scut.edu.cn; 6 School of Fashion Technology, Shanghai University of Engineering Science, Shanghai 201620, China; sunguangwu77@163.com

**Keywords:** photocatalyst, non-solvent induced phase separation, monolith, cellulose, TiO_2_

## Abstract

In this study, novel photocatalyst monolith materials were successfully fabricated by a non-solvent induced phase separation (NIPS) technique. By adding a certain amount of ethyl acetate (as non-solvent) into a cellulose/LiCl/*N*,*N*-dimethylacetamide (DMAc) solution, and successively adding titanium dioxide (TiO_2_) nanoparticles (NPs), cellulose/TiO_2_ composite monoliths with hierarchically porous structures were easily formed. The obtained composite monoliths possessed mesopores, and two kinds of macropores. Scanning Electron Microscope (SEM), Energy Dispersive Spectroscopy (EDS), Fourier Transform Infrared Spectroscopy (FT-IR), X-ray Diffraction (XRD), Brunauer-Emmett-Teller (BET), and Ultraviolet-visible Spectroscopy (UV-Vis) measurements were adopted to characterize the cellulose/TiO_2_ composite monolith. The cellulose/TiO_2_ composite monoliths showed high efficiency of photocatalytic activity in the decomposition of methylene blue dye, which was decomposed up to 99% within 60 min under UV light. Moreover, the composite monoliths could retain 90% of the photodegradation efficiency after 10 cycles. The novel NIPS technique has great potential for fabricating recyclable photocatalysts with highly efficiency.

## 1. Introduction

Wastewater from textile printing containing organic pollutants is one of the main causes of environmental pollution, and thus a threat to both human health and ecosystems [1]. To prevent these contaminants from discharging into the environment, various methods have been developed to directly remove or decompose them.

In recent years, photocatalytic degradation has attracted more and more attention as means in which to remove dyes from wastewater, owing to its green process, and the one-time complete degradation of dyes [2,3]. Titanium dioxide (TiO_2_), one of the most important semiconductors, has been considered as one promising catalyst due to its high optical reactivity, strong oxidation activity, nontoxicity, and low cost. Nevertheless, some inherent shortcomings hinder its further application. On one hand, TiO_2_ nanoparticles are prone to be aggregated, which may reduce the photocatalytic efficiency. On the other hand, the size of TiO_2_ nanoparticles is too small to recycle, which easily causes secondary pollution [4,5,6].

In recent years, the use of photocatalysts supported with hierarchically porous structures has become an effective approach to avoid the aforementioned disadvantages [7,8]. For an ideal photocatalyst support, macropores, mesopores, and micropores are preferred. The macropores are conductive to light capture while the mesopores and micropores facilitate the selection of guest molecules. The reported hierarchically structured porous photocatalyst supports include fibers, membranes, and monoliths [9,10,11]. Among them, hierarchically structured porous polymer monoliths (HSPPMs) have great potential for photocatalyst support. HSPPMs with interconnected hierarchical porosity with a variety of length scales can provide large accessible space, high specific surface area, low density, and high porosity; moreover, compared with other hierarchically porous materials, HSPPMs usually have fast mass transfer and excellent chemical stability.

However, to date, there has been few reports on the fabrication of HSPPMs for photocatalyst support. Xiao and co-workers presented nanoporous polymer monoliths as photocatalyst support for Desussa P25 by solvothermal polymerization [11]. The obtained polymer monoliths exhibited improved photodegradation efficiency. However, they did not mention the stability of polymer monolith under irradiation. As we know, irradiation often impacts polymer stability, which may affect the stability of polymer monoliths as photocatalyst support.

Cellulose is one of the most abundant materials on Earth. Due to its biocompatibility, nontoxicity, biodegradability, and reusability, cellulose has attracted considerable research interests as promising bio-based material candidates to replace petroleum based materials [12]. Furthermore, cellulose is applicable for supporting TiO_2_ nanoparticles owing to its good compatibility with TiO_2_ nanoparticles. Several types of low-dimensional cellulose materials have been reported, including cellulose nanofibers and films by electrospinning and phase separation method, respectively [2,13,14,15,16].

In this study, we report one-pot fabrication of hierarchically porous cellulose/TiO_2_ monoliths by non-solvent induced phase separation (NIPS) method. The NIPS method has been adopted to fabricate a series of HSPPMs [17,18,19]. The obtained cellulose/TiO_2_ monoliths possess interconnected porosity in different length scales, and high specific surface area. Moreover, the cellulose/TiO_2_ monoliths show excellent photocatalytic activity and reusability under UV irradiation. The resultant cellulose/TiO_2_ monoliths in this work have great potentials for wastewater treatment during textile printing, and the technique developed in this paper paves the way toward synthesizing novel photocatalyst supports.

## 2. Results

### 2.1. Fabrication Parameters

**Figure 1:**
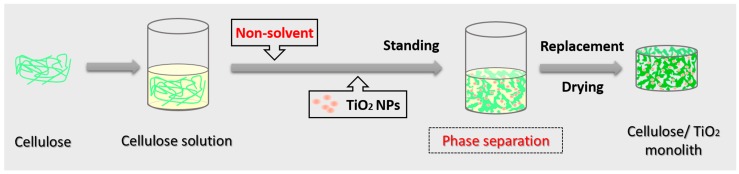
Roadmap for fabricating cellulose/TiO_2_ monoliths. NPs, nanoparticles.

The typical fabrication process is shown as Figure 1. This approach is based on the preparation of cellulose monolith described in our previous work but with some modifications [20]. The ethyl acetate (as the non-solvent) was firstly added into the cellulose solution, a certain amount of TiO_2_ NPs was successively added into the solution system under magnetic stirring. In detail, the amount of cellulose was 85 mg for each cellulose/TiO_2_ monolith, and 1 mg, 10 mg and 20 mg of TiO_2_ NPs were added into cellulose solution, corresponding to the cellulose:TiO_2_ gravimetric ratios of 1:85, 2:17 and 4:17, respectively. The solution with TiO_2_ NPs was then kept for a period of time and phase separation occurred. To guarantee the uniform dispersion of TiO_2_ NPs, the cellulose concentration is a key factor. The cellulose concentration can directly affect both the standing time and the viscosity of the system. The standing times representing the times passing from the addition of the non-solvent to the completion of the phase separation were 1 week, 24 h, 12 h and 3 h for cellulose/TiO_2_ monoliths with the cellulose concentration of 2 wt %, 4 wt %, 6 wt % and 8 wt %, respectively, indicating higher cellulose concentrations lead to shorter standing times. Meanwhile, high cellulose concentrations hinder the dispersion of TiO_2_ NPs. When the concentration was over 4 wt %, the viscosity was too high to prevent the uniform dispersion of TiO_2_ NPs, while when the concentration was lower than 4 wt %, the dispersion of TiO_2_ NPs was much better. Taking into account both the standing time and the dispersion of TiO_2_ NPs, the cellulose concentration of 4 wt % was chosen for the preparation of cellulose/TiO_2_ monoliths.

### 2.2. Scanning Electron Microscope (SEM)

**Figure 2:**
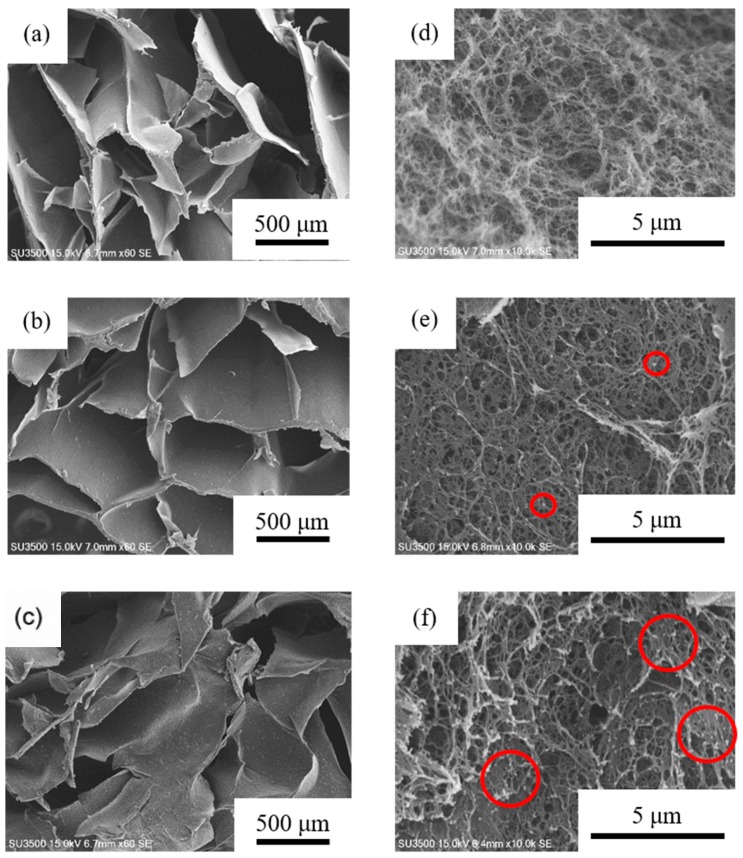
Scanning Electron Microscope (SEM) images of cellulose/TiO_2_ composite monoliths at different magnifications. (**a**): CTM-1; (**b**): CTM-10; (**c**): CTM-20; (**d**): CTM-1; (**e**): CTM-10; (**f**): CTM-20.

The cellulose/TiO_2_ composite monoliths with 1 mg, 10 mg and 20 mg TiO_2_ were fabricated and named as CTM-1, CTM-10 and CTM-20, respectively. Figure 2 displays the micromorphology of CTM-1, CTM-10 and CTM-20. Two kinds of macroporous structures were observed. The macropores with an average pore size of ~500 μm were formed by the sublimation of ice crystals during freeze-drying. Another kind of macropore with an average pore size of ~2.1 μm was formed by phase separation. These macropores are beneficial for the light capture, and may improve the interaction between the TiO_2_ NPs and dyes. In Figure 2a,d, the pore skeleton of CMT-1 has a smooth surface. Higher loading of TiO_2_ results in TiO_2_ aggregation as highlighted in Figure 2e,f.

### 2.3. Nitrogen Adsorption-Desorption Isothems

**Figure 3:**
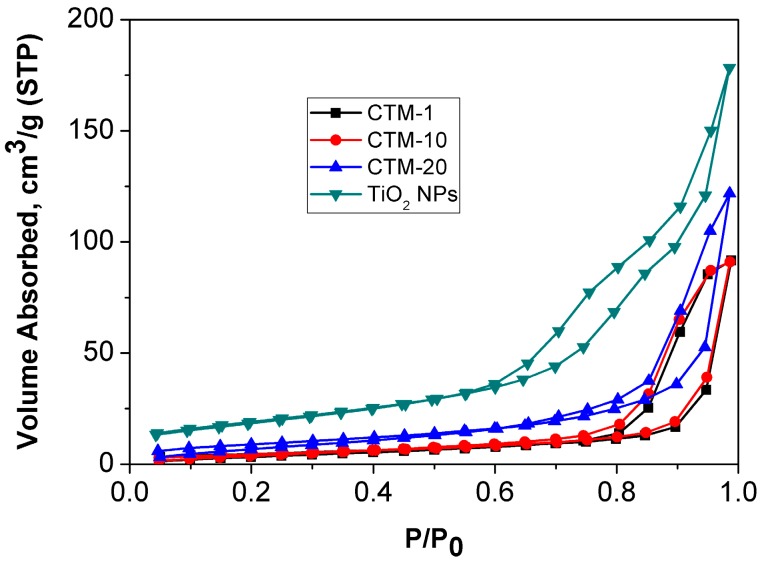
Nitrogen adsorption-desorption isotherms of the cellulose/TiO_2_ monoliths with different TiO_2_ loadings.

The macroporous structure was confirmed by the SEM images (Figure 2), however, it is challenging to detect the mesopores by SEM technique. Here, nitrogen adsorption-desorption characterization was conducted to analyze the mesopores of cellulose/TiO_2_ monoliths. Nitrogen adsorption-desorption isotherms of CTM-1, CTM-10 and CTM-20 are shown in Figure 3. The isotherms of CTM-1, CTM-10 and CTM-20 followed the type II isotherm which was formed by a macroporous absorbent. A typical H3 hysteresis loop in the P/P_0_ ranging from 0.6 to 1.0 was also observed. This hysteresis loop should be caused by capillary condensation, suggesting the existence of slit-like nanoscaled pores in cellulose/TiO_2_ monoliths [21]. The Brunauer-Emmett-Teller (BET) surface areas of CTM-1, CTM-10 and CTM-20 were 16.96, 17.01 and 32.95 m^2^/g, respectively. The increase of BET surface areas is probably due to the increased loading of TiO_2_ NPs leading to higher surface area in the cellulose/TiO_2_ composite monolith.

**Figure 4:**
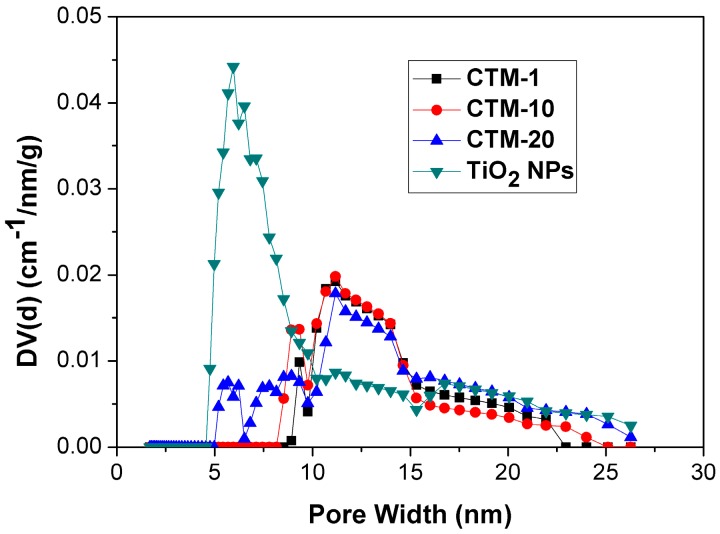
Pore size distribution plots for the cellulose/TiO_2_ monoliths with different TiO_2_ loadings.

The pore size distribution (PSD) plots obtained by the non-local density functional theory (NLDFT) method are shown in Figure 4. The PSDs of CTM-1, CTM-10, and CTM-20 were all centered at ~11.2 nm. There was a slight difference in that the PSD of the composite monolith was wider with increased TiO_2_ NPs loadings. Hence, both BET and PSD results confirm the existence of mesopores in the cellulose/TiO_2_ composite monoliths.

### 2.4. Energy Dispersive Spectrometer (EDS) Analysis

**Figure 5:**
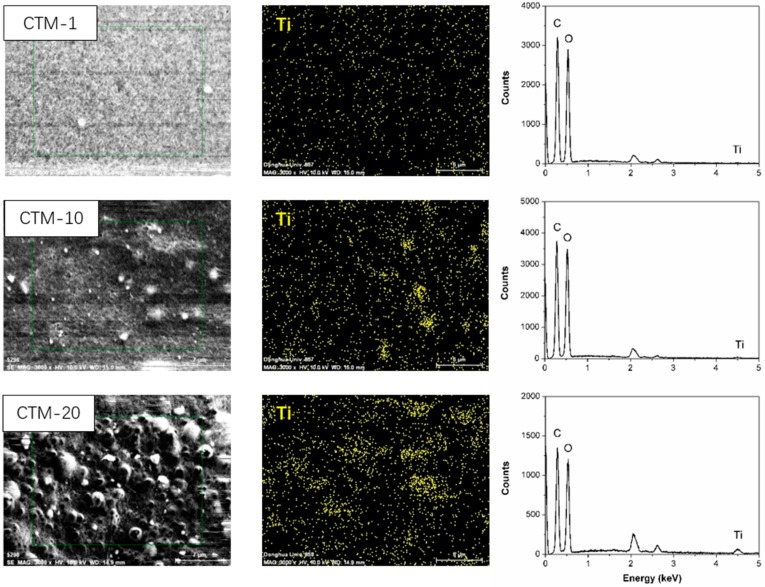
Energy Dispersive Spectrometer (EDS) spectrums of CTM-1, CTM-10 and CTM-20 (from top to bottom).

Figure 5 shows the EDS spectrums of CTM-1, CTM-10 and CTM-20. The pronounced Ti signals on the composite monolith surface confirm the successful decoration of TiO_2_ onto the monolith. The weight percentages of Ti increased from 0.83 wt % to 3.58 wt % as more TiO_2_ was added. Moreover, the dispersion of TiO_2_ was uniform for CTM-1, while TiO_2_ nanoparticles turned to agglomerate with more TiO_2_ nanoparticles.

### 2.5. Fourier Transform Infrared Spectroscopy (FT-IR) Analysis

**Figure 6:**
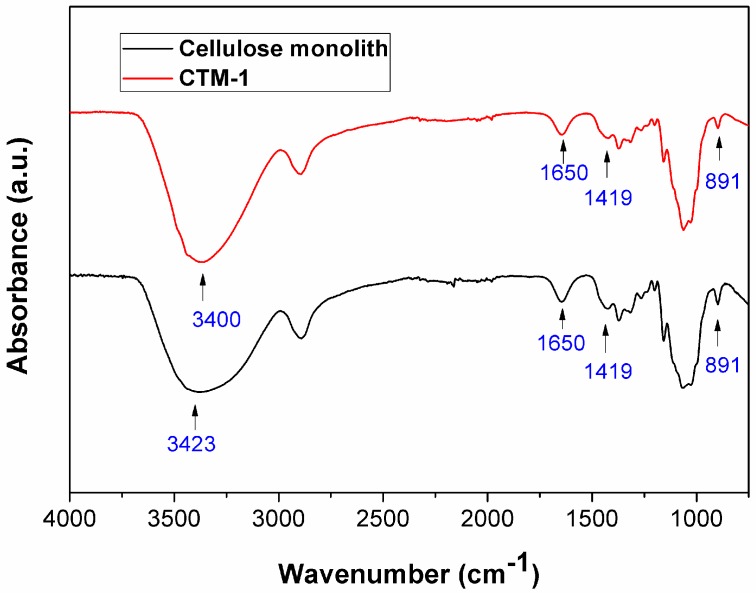
FT-IR spectra of cellulose monolith and CTM-1.

Figure 6 shows the FT-IR spectra of cellulose monolith and CTM-1. As shown in Figure 6, the FT-IR analysis revealed that both the cellulose monolith and CTM-1 showed the characteristic peaks of cellulose II at 891 cm^–1^ and 1419 cm^−1^, respectively. The 891 cm^−1^ peak was assigned to C–O–C stretching at β-linked glucose of cellulose, and the 1419 cm^−1^ band was attributed to CH_2_ symmetry bending [22]. Compared to the cellulose monolith, it was observed that the O–H stretching absorption peak of CTM-1 slightly shifted from 3424 cm^−1^ to 3400 cm^−1^. This is due to the hydrogen bonding interactions between the O–H groups of cellulose and Ti–O bond of TiO_2_ [23]. It is noteworthy that the O–H stretching absorption peak of CTM-1 was sharper in comparison with that of cellulose monolith. This further proves interfacial interactions between TiO_2_ and cellulose. The possible mechanism is that the Ti–OH groups on the surface of TiO_2_ react with the hydroxyl groups in the cellulose chain, forming hydrogen bonding on the inner and outer surfaces of CTM matrix [13].

### 2.6. X-ray Diffraction (XRD) Analysis

**Figure 7:**
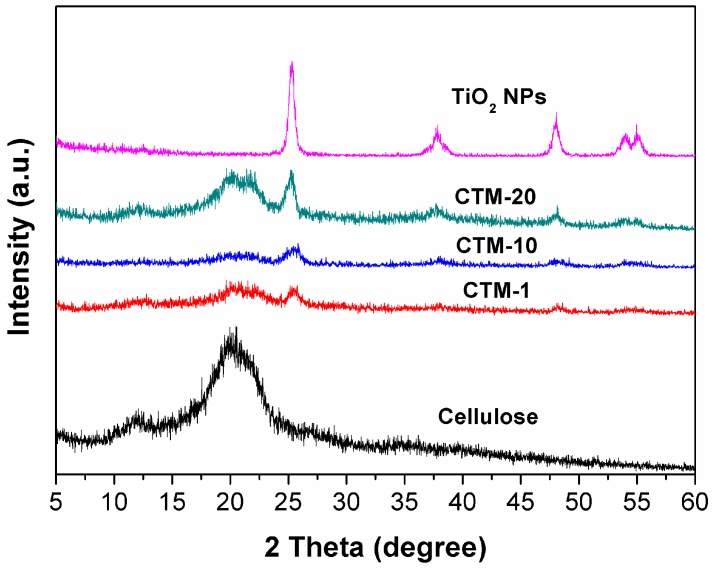
XRD patterns of cellulose monolith, cellulose/TiO_2_ monoliths, and TiO_2_ NPs.

Figure 7 shows the XRD patterns of TiO_2_ NPs, cellulose monolith, and cellulose/TiO_2_ monoliths with different TiO_2_ amounts. The peaks at 2θ = 12.1°, 20.3° and 21.8° corresponded to the ($1\bar{1}0$), (110), and (200) planes of cellulose II crystalline, respectively [24], which was in accordance with FT-IR analysis. Anatase TiO_2_ possessed its characteristic peaks at 25.46°, 37.78°, 47.96° and 54.14°, indicating the successful incorporation of anatase TiO_2_ NPs into porous cellulose monolith matrix [14]. Compared to controlled cellulose monolith, the peaks of CTM-1 assigned to cellulose II crystalline were significantly quenched. It is probably due to the fact that the crystalline pattern of cellulose formed by hydrogen bonding between hydroxyl groups of cellulose chains is disrupted by the further addition of TiO_2_. Moreover, the peak positions of CTM-1 lightly shifted to the right compared to the peaks for the cellulose monolith without TiO_2_ NPs loading, proving the occurrence of hydrogen interactions between TiO_2_ NPs and cellulose.

### 2.7. X-ray Photoelectron Spectroscopy (XPS) Analysis

**Figure 8:**
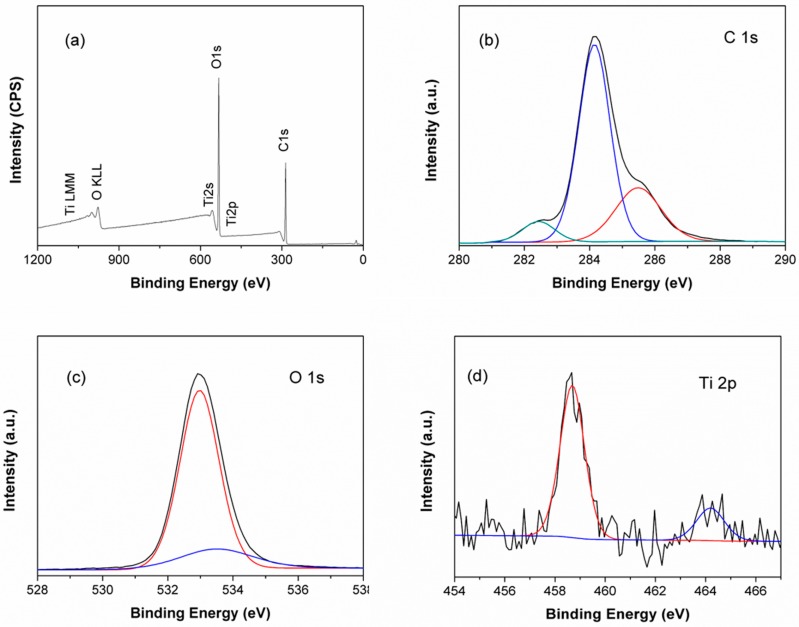
(**a**) X-ray photoelectron spectroscopy (XPS) spectra of the CTM-1 sample and high-resolution XPS spectra of (**b**) C 1s; (**c**) O 1s; and (**d**) Ti 2p.

Chemical surface characterization of CTM-1 was studied by XPS. In Figure 8a, the peaks of Ti 2p, Ti 2s, Ti 3s, Ti 3p, O 1s, and C 1s were observed in the spectra. The 981.3 and 1113.1 eV peaks represent the Auger electron peaks of O and Ti, respectively. Figure 8b shows the high resolution of C 1s peak in the range of binding energy from 280 to 290 eV. The peaks at 284.2 and 285.2 eV were assigned to (C–C–OH boning) and (O–C–O or C=O bonding) respectively, revealing that the carbon atoms come from cellulose molecules [25]. Furthermore, the peak at 282.4 eV confirms the existence of C as a dopant in TiO_2_ photocatalyst, since the doped carbon in TiO_2_ showed a very low C 1s binding energy at 281–282 eV [26]. Figure 8c shows the high resolution XPS spectra of O 1s. Two peaks centered at 532.9 eV and 533.4 eV correspond to the oxygen atoms in alcohol groups. The two distinct peaks at 458.7 eV (Ti 2p_3/2_) and 464.7 eV (Ti 2p_1/2_) correspond to Ti^4+^ in pure anatase in Figure 8d [27].

### 2.8. Ultraviolet-Visible Spectroscopy (UV-Vis) Transmission Measurement

**Figure 9:**
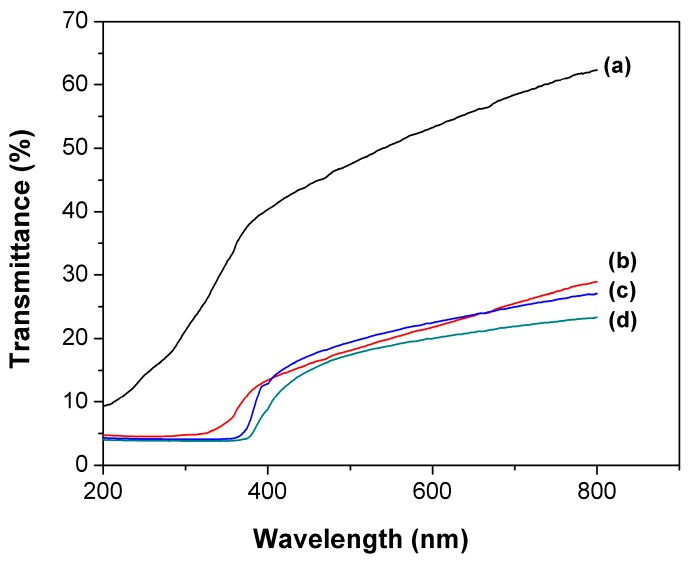
Optical transmission spectra of (**a**) 2 mm thick CTM-1; (**b**) 3.5 mm thick CTM-1; (**c**) 3.5 mm thick CTM-10; and (**d**) 3.5 mm thick CTM-20.

As a photocatalyst support, the photopermeability is another key factor, since it can directly affect the photocatalyst activity. Due to its 3-dimensional shape on a macro level, the thickness of the cellulose/TiO_2_ composite monolith impacts the permeability of UV light. Therefore, we measured the photopermeability of CTM-1 with a thickness of 2 mm and 3.5 mm, respectively. As seen in Figure 9a,b, the 2 mm thick CTM-1 demonstrated higher transmittance of ~70%, compared with that of 3.5 mm thick CTM, which has a lower transmittance of ~30%. Additionally, the photopermeabilities of CTM-10 and CTM-20 with a thickness of 3.5 mm were 26% and 23%, respectively, indicating that the photopermeability was slightly decreased with an increase in TiO_2_ amount. These results clearly revealed that the thickness of cellulose/TiO_2_ composite monolith strongly affects the transmittance of UV light. Thus, we selected 2 mm thick samples for the future photocatalytic experiments.

### 2.9. Photocatalytic Activity

**Figure 10:**
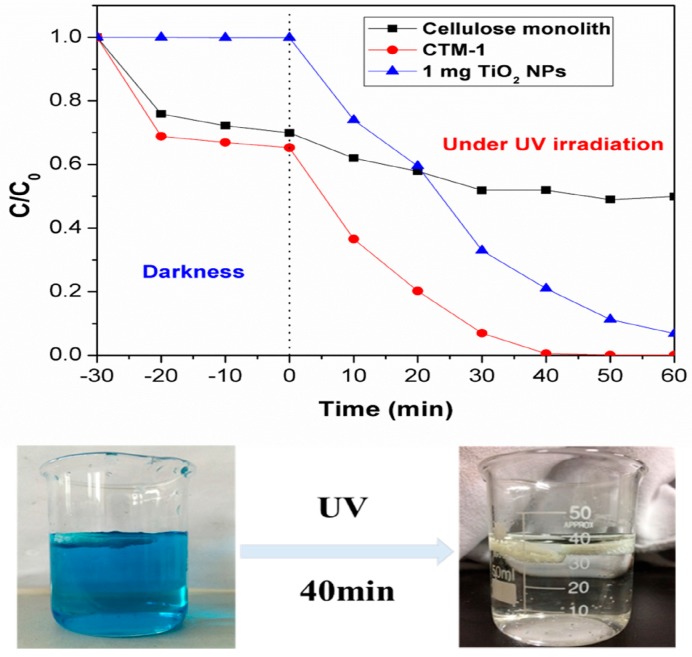
Photocatalytic degradation rate of methylene blue (MB) over the different samples under UV light.

To investigate the potential photocatalytic ability of cellulose/TiO_2_ composite monolith, the photodegradations of methylene blue (MB) aqueous solution under UV light in the presence of CTM-1, cellulose monolith, and TiO_2_ NPs were monitored by UV-vis spectrometry (Figure 10). The amounts of cellulose and TiO_2_ NPs for CTM-1 in this experiment were 85 mg and 1 mg, respectively, and the cellulose monolith was of 85 mg. Furthermore, 1 mg of TiO_2_ NPs without loading was also used. Before the irradiation process, the samples were immersed in the MB solution in the dark for ~30 min. The monoliths were transformed from white to nattier blue after soaking in the MB solution, and the adsorption rates for cellulose monolith and CTM-1 were 30% and 34%, respectively. The TiO_2_ NPs exhibited neglectable adsorptive properties. Meanwhile, the solution color was still blue. After a 60 min irradiation, the color removal of the dye solution was complete in the presence of CTM-1. The degradation rate was higher than 99% for the composite monolith. In contrast, TiO_2_ NPs resulted in a degradation rate of ~90%. It was worth noting that after 30 min irradiation, the dye was almost removed with a photodegradation of ~90% in the dye solution containing composite monolith, while there was only a 70% degradation of dye for the TiO_2_ NPs. The higher efficiency for the cellulose/TiO_2_ composite monolith is probably because of the degradation of MB molecules adsorbed on the cellulose/TiO_2_ composite monolith and the readsorption of MB in the solution. The adsorptive property of the composite monolith caused by hierarchically porous structures improves the frequency of contact between photocatalysts and organic pollutants [11].

### 2.10. Photostability Measurements

**Figure 11:**
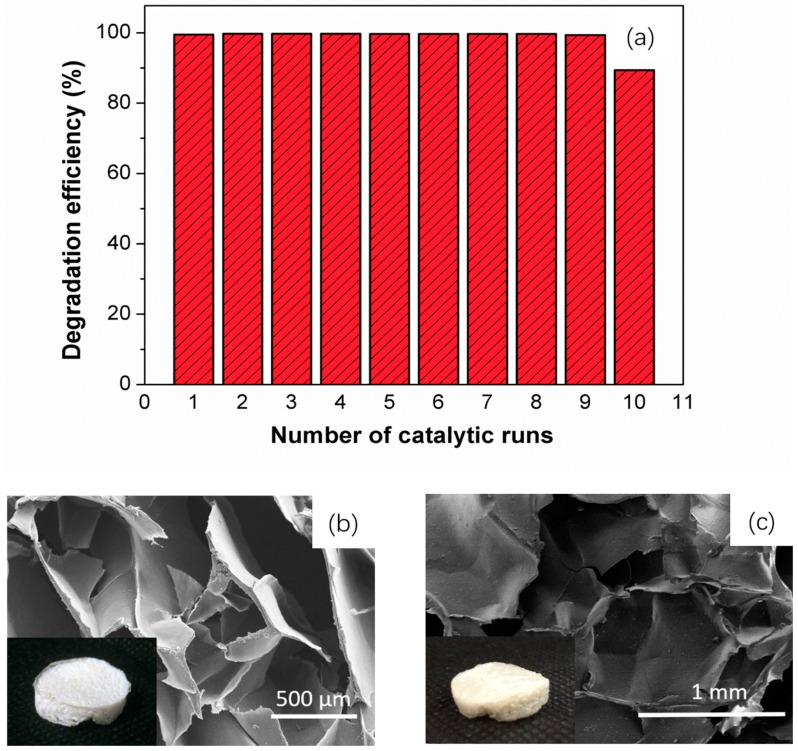
(**a**) Degradation efficiency of a methylene blue solution (120 ppm) after successive reuse of CTM-1 catalyst and the morphology of (**b**) CTM-1 before and (**c**) CTM-1 after 10 cycles as a photocatalyst.

The stability of photocatalyst is essential for practical application. To evaluate the stability of the catalyst, the CTM-1 showing the best efficiency in the photocatalytic process was adopted for 10 cycles in 60 min (Figure 11a). The photocatalytic activity was retained after 10 cycles, and showed a high removal efficiency of ~99 for 9 cycles, and 90 for 10 cycles. The shape and microstructure of CTM-1 after 10 cycles are shown as Figure 11c. Compared to the microstructure of CTM-1 before using it as a photocatalyst (Figure 11b), the porous structure of CTM-1 after 10 cycles was almost retained. On the basis of these results, it was proven to be a potential reusable catalyst.

A comparison for the photocatalytic degradation activity of MB between cellulose/TiO_2_ monolith and other systems is shown as Table 1. Compared with other systems, cellulose/TiO_2_ monolith possessed not only a higher photodegradation rate in a shorter reaction time, but also excellent reusability.

## 3. Discussion

**Scheme 1:**
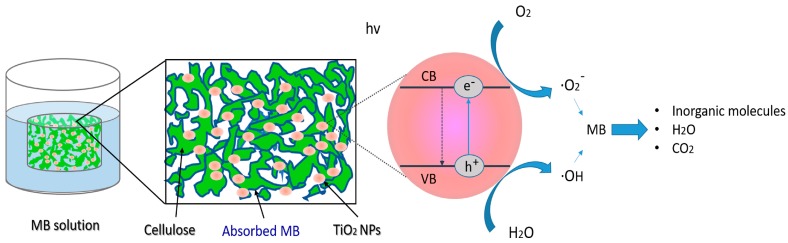
The schematic representation of the photocatalytic mechanism.

Scheme 1 shows the scheme of the photocatalytic mechanism. The TiO_2_ NPs is well dispersed into the solution system during the fabrication process. On one hand, TiO_2_ interacts with cellulose through hydrogen bonds; on the other hand, the physical space blocks from cellulose can prevent the aggregation of TiO_2_ as well. Moreover, during the adsorption process, ~10% of MB can be adsorbed onto the surface of the composite monolith due to the hierarchically porous structure, and it improves the interaction between TiO_2_ and the dye, at the same time, the macropores with an average pore size over 500 μm are feasible for the capture of the light, both of which can improve the photocatalytic activity.

## 4. Materials and Methods

### 4.1. Materials

Microcrystalline cellulose powder from cotton linters and lithium chloride (LiCl) were purchased from Sigma Aldrich Company, Saint Louis, MO, USA. Methylene blue, *N*,*N*-Dimethylacetamide (DMAc) and methanol were purchased from Shanghai Lingfeng Chemical Reagent Co., Ltd., Shanghai, China. Titanium oxide nanoparticles (TiO_2_ NPs) (99.8% metal basis anatase, 40 nm, hydrophilic) were purchased from Shanghai Aladdin Bio-Chem Technology Co., Ltd., Shanghai, China. Ethyl acetate, acetone, and other reagents were used as received.

### 4.2. Preparation of Cellulose Solution

Microcrystalline cellulose powder was firstly dissolved in LiCl (8 wt %)-DMAc solution to get a cellulose solution with a concentration of 4 wt %, according to the reported work of Zhang [31]. Briefly, cellulose powder was activated by distilled water, methanol, and DMAc for 4 h, 2 h and 2 h, respectively. Subsequently, the activated cellulose powder was added into the LiCl/DMAc solution with a concentration of 8 wt % under N_2_ protection. The system was stirred at room temperature until the activated cellulose powder was dissolved completely, and then the cellulose solution was incubated at 4 °C for 12 h, and the stable cellulose solution was thus formed.

### 4.3. Preparation of Cellulose/TiO_2_ Monoliths

Firstly, 1 mL of ethyl acetate as non-solvent was added dropwise into 2 mL of cellulose solution with a cellulose concentration of 4 wt % under stirring at room temperature. The adding procedure was slow in order to avoid the precipitation of cellulose. Secondly, TiO_2_ nanoparticles with different amounts were added into the solution system under stirring. The mixed system was then kept for 24 h, where phase separation occurred and a white gel was formed. Afterwards, the gel was replaced by acetone at least three times to remove any solvent. Finally, the cellulose/TiO_2_ monolith was obtained after freeze-drying.

### 4.4. Characterizations

The microstructure of cellulose/TiO_2_ monoliths was observed with a Scanning Electron Microscope (SEM, S-3000N, Hitachi Ltd., Tokyo, Japan) at 15 kV. Before observation, the samples were cut with a scalpel and coated with a thin layer of gold by an ion sputter apparatus (E-1010 Ion Sputter, Hitachi Ltd., Tokyo, Japan). The specific surface area of the cellulose monolith was measured by a nitrogen adsorption apparatus using the Brunauer-Emmett-Teller (BET) method (NOVA 4200e, Quantachrome Instruments, Boynton Beach, FL, USA). Energy Dispersive Spectroscopy (EDS, Quantax 400, Bruker, Germany) was adopted to investigate the chemical composition of the composite monolith surface. The chemical structure of the composite monolith was analyzed using a Nicolet Is 5 Fourier transform infrared spectrophotometer. The UV-Vis adsorption spectra were carried out using a Shimadzu UV-2550 spectrophotometer. XRD patterns were run on an X-ray diffractometer (Rigaku D/max-2550 VB/PC, RICOH Company, Ltd., Tokyo, Japan) at a scanning rate of 4.0°/min from 5° to 60° (2θ). The measurement was performed under conditions of 40 kV and 30 mA using Cu-Kα radiation (λ = 1.5418 Å). The surface chemical composition of the samples was analyzed by X-ray photoelectron spectroscopy (XPS, AXIS ULTRA DLD, Kratos Analytical Ltd., Manchester, UK) with an Al K_α_ radiation source.

### 4.5. Photocatalytic Degradation of Methylene Blue (MB)

The photocatalytic activity of the as-prepared cellulose/TiO_2_ composite monolith was evaluated by observing the degradation of MB dye solution under UV irradiation. Typically, the cellulose/TiO_2_ composite monoliths with different TiO_2_ amounts (1 mg, 10 mg and 20 mg) were immersed into 20 mL of MB aqueous solution (~12 ppm), respectively. The mixture was placed in the dark for 30 min to ensure complete adsorption of MB dye. Then, the solution containing photocatalysts was exposed to the UV irradiation by a 125 W mercury lamp for another 1 h to evaluate the photocatalytic activity. The concentration of MB was measured at every 10 min by recording its absorbance at 665 nm wavelength with a UV-visible spectrophotometer (UV-2550 spectroscopy system, Shimadzu, Tokyo, Japan), from which the degradation efficiency was calculated. The process was repeated several times until the photocatalytic activity was obviously decreased.

## 5. Conclusions

Cellulose/TiO_2_ composite monoliths have been successfully prepared using a simple NIPS method. Ethyl acetate was chosen as the non-solvent to induce the phase separation of cellulose solution containing TiO_2_ NPs, and the composite monoliths could be easily fabricated after solvent exchange and freeze-drying. SEM images of the obtained monoliths revealed two kinds of interconnected macropores existing in the composite monoliths. Relatively large surface area and mesopores were confirmed by BET measurement. The cellulose/TiO_2_ composite monolith exhibited high photocatalytic activity. This technique has great potential for the fabrication of high-performance photocatalysts for water treatment.

## Figures and Tables

**Table 1:** Photocatalytic degradation activity of MB over different systems under UV light.

Type	Degradation Rate	Degradation Time (min)	Reusability	Ref.
TiO_2_/Polymer nanofibers	~70%	180	4	[13]
TiO_2_-cellulose nanofiber films	~75%	60	5	[28]
Niobium oxyhydroxide dispersed Poly(3-hydroxybutyrate) films	~92%	120	7	[29]
Nanocrystalline ZnO/cellulose	98.3%	180	5	[30]
TiO_2_/cellulose monolith	99%	40	9	-

## References

[1] Konstantinou I.K., Albanis T.A. (2004). TiO_2_-assisted photocatalytic degradation of azo dyes in aqueous solution: kinetic and mechanistic investigations—A review. Appl. Catal. B Environ..

[2] Jin X., Xu J., Wang X., Xie Z., Liu Z., Liang B., Chen D., Shen G. (2014). Flexible TiO_2_/cellulose acetate hybrid film as a recyclable photocatalyst. RSC Adv..

[3] Pelaez M., Nolanb N.T., Pillai S.C., Seery M.K., Falaras P., Kontos A.G., Dunlop P.S.M., Hamilton J.W.J., Byrne J.A., O’Shea K. (2012). A review on the visible light active titanium dioxide photocatalysts for environmental applications. Appl. Catal. B Environ..

[4] Postnova I., Kozlova E., Cherepanova S., Tsybulya S., Rempel A., Shchipunov Y. (2015). Titania synthesized through regulated mineralization of cellulose and its photocatalytic activity. RSC Adv..

[5] Lee S.-Y., Park S.-J. (2013). TiO_2_ photocatalyst for water treatment applications. J. Ind. Eng. Chem..

[6] Liu N., Chen X., Zhang J., Schwank J.W. (2014). A review on TiO_2_-based nanotubes synthesized via hydrothermal method: Formation mechanism, structure modification, and photocatalytic applications. Catal. Today.

[7] Gu S., Xie J., Li C.M. (2014). Hierarchically porous graphitic carbon nitride: Large-scale facile synthesis and its application toward photocatalytic dye degradation. RSC Adv..

[8] Li M., Li X., Jiang G., He G. (2015). Hierarchically macro-mesoporous ZrO_2_-TiO_2_ composites with enhanced photocatalytic activity. Ceram. Int..

[9] Han C., Wang Y., Lei Y., Wang B. (2016). Modification of hierarchically porous SiC ultrafine fibers with tunable nitrogen-containing surface. Ceram. Int..

[10] Ognibene G., Cristaldi D.A., Fiorenza R., Blanco I., Cicala G., Scire S., Fragala M.E. (2016). Photoactivity of hierarchically nanostructured ZnO-PES fibre mats for water treatments. RSC Adv..

[11] Zhang Y., Wei S., Zhang H., Liu S., Nawaz F., Xiao F.S. (2009). Nanoporous polymer monoliths as adsorptive supports for robust photocatalyst of Degussa P25. J. Colloid Interface Sci..

[12] Klemm D., Heublein B., Fink H.P., Bohn A. (2005). Cellulose: Fascinating biopolymer and sustainable raw material. Angew. Chem. Int. Ed..

[13] Abdal-hay A., Makhlouf A.S., Khalil K.A. (2015). Novel, Facile, Single-Step Technique of Polymer/TiO(2) Nanofiber Composites Membrane for Photodegradation of Methylene Blue. ACS Appl. Mater. Interfaces.

[14] Morawski A.W., Kusiak-Nejman E., Przepiórski J., Kordala R., Pernak J. (2013). Cellulose-TiO_2_ nanocomposite with enhanced UV–Vis light absorption. Cellulose.

[15] Wittmar A., Thierfeld H., Köcher S., Ulbricht M. (2015). Routes towards catalytically active TiO_2_ doped porous cellulose. RSC Adv..

[16] Wittmar A., Vorat D., Ulbricht M. (2015). Two step and one step preparation of porous nanocomposite cellulose membranes doped with TiO_2_. RSC Adv..

[17] Sun X., Fujimoto T., Uyama H. (2013). Fabrication of a poly(vinyl alcohol) monolith via thermally impacted non-solvent-induced phase separation. Polym. J..

[18] Sun X., Sun G., Wang X. (2017). Morphology modeling for polymer monolith obtained by non-solvent-induced phase separation. Polymer.

[19] Xin Y., Xiong Q., Bai Q., Miyamoto M., Li C., Shen Y., Uyama H. (2017). A hierarchically porous cellulose monolith: A template-free fabricated, morphology-tunable, and easily functionalizable platform. Carbohydr. Polym..

[20] Sun X., Wang K., ZHANG B.-X., Zou F., Sun G., Han W., Wang X. (2017). Hierarchically porous cellulose monolith prepared by combination of an ice-template method and a non-solvent induced phase separation method. Chem. Lett..

[21] Sing K.S.W., Everett D.H., Haul R.A.W., Moscou L., Pierotti R.A., Rouquerol J., Siemieniewska T. (1985). Reporting physisorption data for gas/solid systems. Pure Appl. Chem..

[22] Mohamed M.A., Salleh W.N., Jaafar J., Ismail A.F., Mutalib M.A., Sani N.A.A., Asri S.E.A., Ong C.S. (2016). Physicochemical characteristic of regenerated cellulose/N-doped TiO_2_nanocomposite membrane fabricated from recycled newspaper with photocatalytic activity under UV and visible light irradiation. Chem. Eng. J..

[23] Zeng J.L., Cai J., Zhang L. (2010). TiO_2_ immobilized in cellulose matrix for photocatalytic degradation of phenol under weak UV light irradiation. J. Phys. Chem..

[24] Wada M., Sugiyama J., Okano T. (2003). Native celluloses on the basis of two crystalline phase (Iα/Iβ) system. J. Appl. Polym. Sci..

[25] Pereira P.H.F., Voorwald H.J.C., Cioffi M.O.H., da Silva M.L.C.P., Rego A.M.B., Ferraria A.M., de Pinho M.N. (2014). Sugarcane bagasse cellulose fibres and their hydrous niobium phosphate composites: Synthesis and characterization by XPS, XRD and SEM. Cellulose.

[26] Mohamed M.A., Salleh W.N.W., Jaafar J., Ismail A.F. (2015). Structural characterization of N-doped anatase-rutile mixed phase TiO_2_ nanorods assembled microspheres synthesized by simple sol-gel method. J. Sol-Gel Sci. Technol..

[27] Sim L.C., Tan W.H., Leong K.H., Bashir M.J.K., Saravanan P., Surib N.A. (2017). Mechanistic characteristics of surface modified organic semiconductor g-C_3_N_4_ nanotubes alloyed with titania. Materials.

[28] Snyder A., Bo Z., Moon R., Rochet J.C., Stanciu L. (2013). Reusable photocatalytic titanium dioxide-cellulose nanofiber films. J. Colloid Interface Sci..

[29] Heitmann A.P., Patrício P.S.O., Coura I.R., Pedroso E.F., Souza P.P., Mansur H.S., Mansur A., Oliveira L.C.A. (2016). Nanostructured niobium oxyhydroxide dispersed Poly (3-hydroxybutyrate) (PHB) films: Highly efficient photocatalysts for degradation methylene blue dye. Appl. Catal. B Environ..

[30] Zuo H.-F., Guo Y.-R., Li S.-J., Pan Q.-J. (2014). Application of microcrystalline cellulose to fabricate ZnO with enhanced photocatalytic activity. J. Alloys Compd..

[31] Zhang B.-X., Azuma J.-I., Uyama H. (2015). Preparation and characterization of a transparent amorphous cellulose film. RSC Adv..

